# Multiple stark juckende erythrosquamöse Plaques

**DOI:** 10.1007/s00105-026-05700-7

**Published:** 2026-05-18

**Authors:** Lea-Isabell Präger, Marie Rabe, Manfred Kunz, Pietro Nenoff, J. C. Simon

**Affiliations:** 1 Klinik und Poliklinik für Dermatologie, Venerologie und Allergologie, Universitätsklinikum Leipzig, Philipp-Rosenthal-Straße 23, 04103 Leipzig, Deutschland; 2 Laboratory for Medical Microbiology, Mölbis, Deutschland

**Keywords:** Mykose, Infektiologie, Mycosis, Infectious diseases

## Abstract

Wir berichten über eine 26-jährige schwangere Patientin, die sich mehrfach notfallmäßig aufgrund von stark juckenden Hautbefunden in unserer Klinik vorstellte. Auch der Partner und ihre 2 Kinder waren von ähnlichen Hautveränderungen betroffen. In der mykologischen Diagnostik konnte *Trichophyton indotineae* nachgewiesen werden. Dieser Erreger breitet sich ausgehend vom indischen Subkontinent auch in Europa zunehmend aus. Aufgrund von Resistenzen gegen Terbinafin stellt die Behandlung eine Herausforderung dar. An diesem Fallbeispiel werden Diagnostik und Therapiebesonderheiten diskutiert.

## Abstract (en)

We report on a 26-year-old pregnant woman who presented to our clinic due to severely itchy skin lesions. Her partner and her 2 children were also affected by similar skin lesions. *Trichophyton indotineae* was detected in polymer chain reaction (PCR) tests. This pathogen is increasingly spreading in Europe, originating from the Indian subcontinent. Treatment poses a challenge due to resistance to terbinafine. This case study discusses diagnostic and therapeutic considerations.

## Überblick

Wir berichten über eine 26-jährige schwangere Patientin, die sich mehrfach notfallmäßig aufgrund von stark juckenden Hautbefunden in unserer Klinik vorstellte.

## Fallbericht

**Abb. 1:**
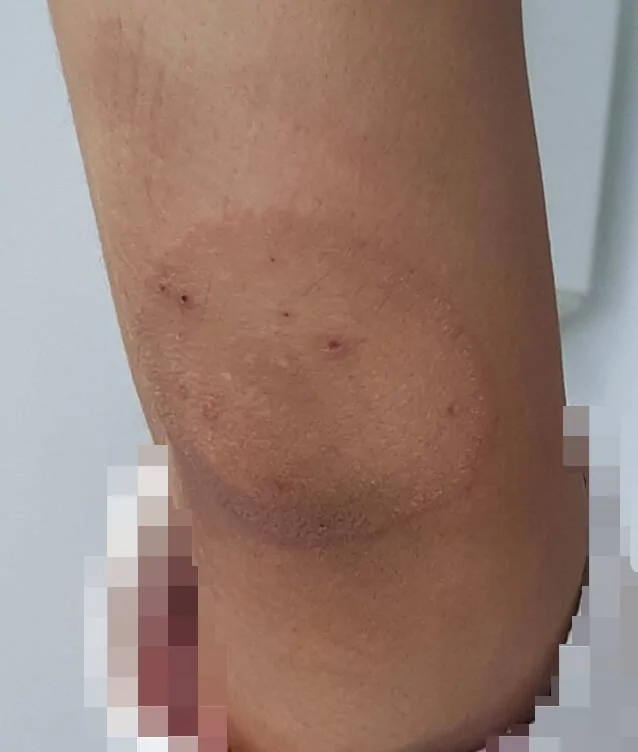
Kreisrunde randbetonte hyperpigmentierte schuppende Plaque am Ellenbogen

Unsere 26-jährige syrische Patientin berichtet über stark juckende, erythematöse, randbetont schuppende, bis zu 20 cm durchmessende Papeln und Plaques an Armen, Stamm, medialen Oberschenkeln und Gesäß (Abb. 1, 2 und 3). Die Patientin befand sich zum Zeitpunkt der Erstvorstellung in der 6. Schwangerschaftswoche. Der Partner und 2 Kinder im Alter von 3 und 5 Jahren sowie die Familie des Bruders hatten ähnliche Hautveränderungen. Die Befunde bestanden seit 7 Monaten. Bei klinischem Verdacht auf eine Tinea corporis wurden bereits multiple Lokal- und Systemtherapien angewandt: Miconazol, Betamethason-Dipropionat und Clotrimazol bei der Patientin und den Kindern, sowie Terbinafin und Fluconazol oral beim Partner. Jedoch kam es zu keinem zufriedenstellenden Ansprechen.

**Abb. 2:**
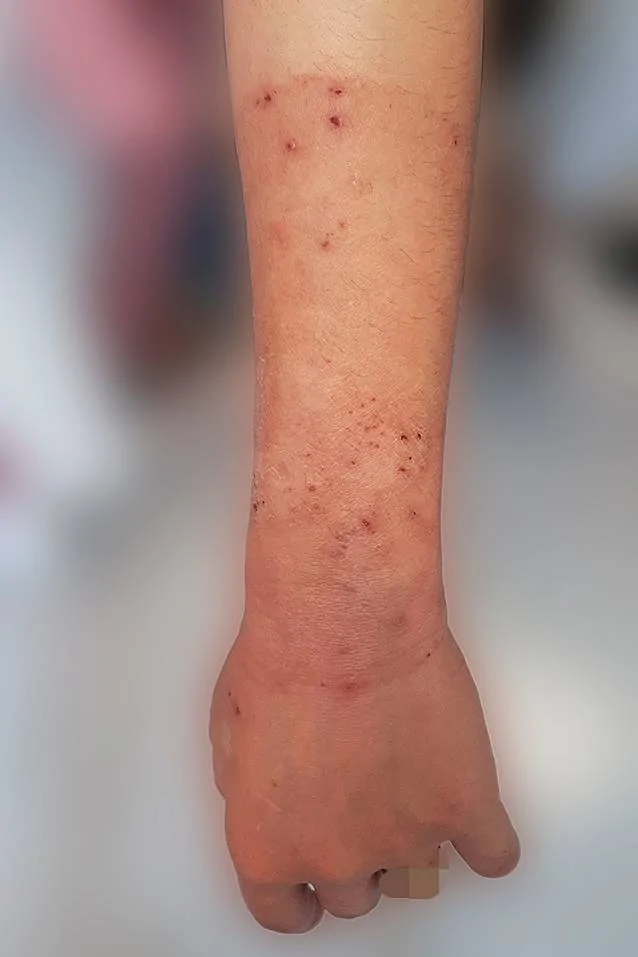
Kreisrunde randbetonte hyperpigmentierte schuppende Plaque mit deutlichen Kratzexkoriationen am Unterarm

**Abb. 3:**
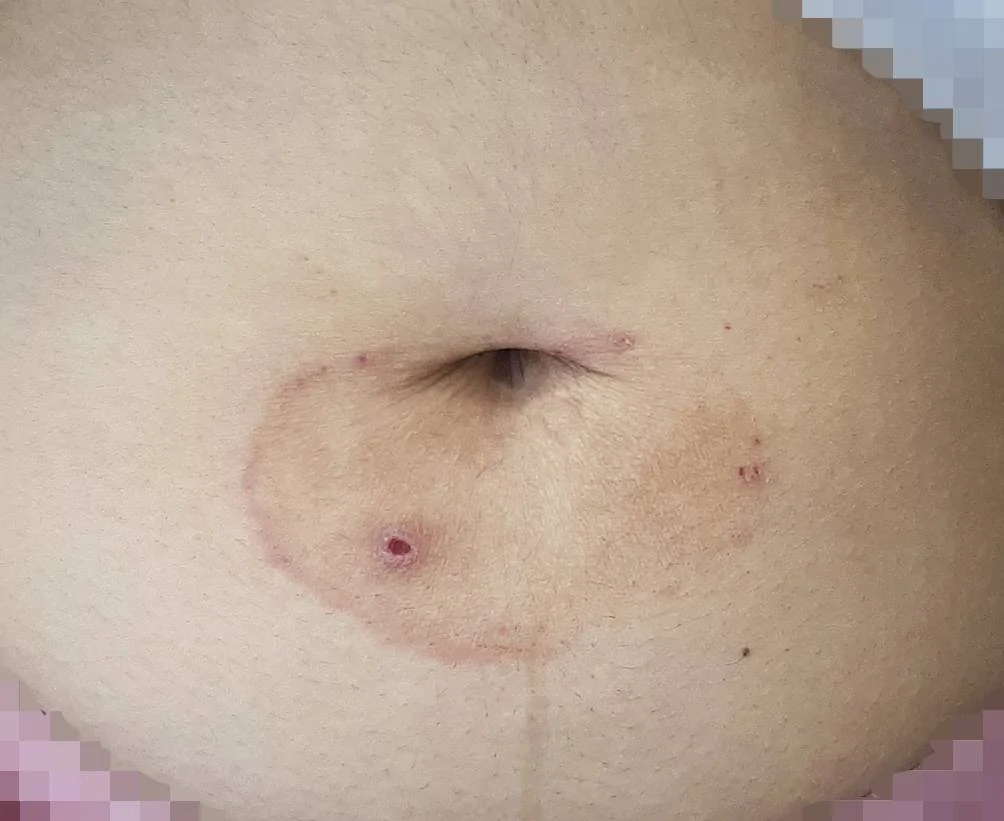
Identische Läsion wie Abb. 1 und Abb. 2 am Bauchnabel

## Diagnose

**Diagnose:** Tinea corporis durch *Trichophyton indotineae* mit Terbinafin-Resistenz

## Diagnostik und klinischer Verlauf

Wir entnahmen Hautschuppen vom linken Oberschenkel zur mykologischen Diagnostik. Mittels PCR konnte ein Erreger des *Trichophyton* (T.)-*mentagrophytes/interdigitale*-Komplexes nachgewiesen werden. Die Identifizierung von *Trichophyton indotineae* (T. mentagrophytes ITS Genotyp VIII) erfolgte im Verlauf mittels Kultur und nachfolgender Sequenzierung der ITS(„internal transcribed spacer“)-Region der fungalen rDNA. Die Empfindlichkeitstestung dieses Stammes gegenüber Terbinafin mittels SQLE(Squalenepoxidase)-Genmutationsanalyse sowie Agardilutionstest ergab eine Terbinafin-Resistenz sowie Sensibilität gegenüber Itraconazol.

Aufgrund der bestehenden Schwangerschaft wäre eine antimykotische Systemtherapie nur nach ausführlicher Nutzen-Risiko-Abwägung im Off-Label-Bereich möglich gewesen. Dies lehnte die Patientin ausdrücklich ab. Daher behandelten wir ausschließlich topisch mit Clotrimazol 1 % für insgesamt 8 Wochen zweimal täglich. Unter dieser Therapie konnte eine deutliche Reduktion der Hautbefunde, jedoch keine vollständige Abheilung erzielt werden. Im Konsens mit der Patientin wurden eine konsequente Fortführung der topischen Therapie bis zum Ende der Schwangerschaft und eine anschließende Systemtherapie mit Itraconazol beschlossen.

Der Partner zeigte unter topischem Clotrimazol 1 % und Ciclopirox 1 % je einmal täglich sowie Itraconazol 100 mg oral zweimal täglich nach 8 Wochen ein vollständiges Therapieansprechen.

Bei den Kindern kam es unter Bifonazol 1 % Creme über 8 Wochen an den betroffenen Stellen zur vollständigen Abheilung der Hautbefunde.

## Diskussion

Die Taxonomie des *T. mentagrophytes/T. interdigitale*-Komplexes hat sich seit 2007 immer wieder verändert. *Trichophyton indotineae I* wurde von Nenoff et al. zunächst als *T. mentagrophytes *ITS-Genotyp *VIII *beschrieben [1]. Seit 2020 wird dieser neue Genotyp aufgrund seiner klinisch signifikanten Eigenschaften und der Terbinafin-Resistenz, die mehr als 60 % der Stämme *in vitro* und *in vivo* aufweisen, als eigene Spezies klassifiziert [2].

In den letzten Jahren haben sich therapierefraktäre Dermatophytosen durch *T*. *indotineae* vorrangig in Südostasien – insbesondere auf dem indischen Subkontinent – epidemisch ausgebreitet. Inzwischen hat sich der Erreger in Indien so fest etabliert, dass er andere Dermatophyten weitgehend verdrängt hat und nun die dominante, konstant präsente Spezies darstellt, weshalb einige Autoren von einer Endemie sprechen [3]. Zunehmend wird jedoch ein Auftreten des neuen Erregers in Ländern des Nahen Ostens und Europas beobachtet [4].

Die Infektion erfolgt vor allem als Mensch-zu-Mensch-Übertragung. Jedoch sind auch wenige Tiere als Infektionsquelle beschrieben [4]. Mittlerweile wurden in Deutschland bei zahlreichen Patienten Isolate nachgewiesen; überwiegend handelte es sich um Personen mit südasiatischem oder arabischem Hintergrund, während Infektionen bei deutschstämmigen Patienten seltener sind [5]. Typisch sind kreisrunde entzündliche hyperpigmentierte schuppende doppeltkonturierte randbetonte und sich zentrifugal ausbreitende Plaques mit Lokalisationen am Genitale, inguinal, am Körperstamm und im Gesicht mit ausgeprägtem Juckreiz [5, 6]. Diese stellen sich oft als therapierefraktär heraus [5].

Missense-Mutationen des Gens der Squalenepoxidase sind verantwortlich für die Resistenz gegenüber Terbinafin [7]. In einer indischen Studie waren 76 % der *T.-indotineae*-Infektionen resistent gegenüber topischem und oralem Terbinafin [8]. Resistenzen wurden in vitro zudem gegen Fluconazol und Griseofulvin nachgewiesen [4]. Die Resistenzbildung steht wahrscheinlich im Zusammenhang mit dem unkritischen Gebrauch von Kombinationspräparaten aus hochpotenten Steroiden, Antimykotika und Antibiotika, welche in Indien und anderen Ländern des globalen Südens rezeptfrei erhältlich sind [4]. Vermutlich wird hierdurch das Hautmikrobiom und das lokale Immunsystem ungünstig beeinflusst.

Die Therapie der Wahl für eine Dermatophytose durch *T. indotineae* stellt Itraconazol 100 mg zweimal täglich über 4–8 Wochen dar. In Einzelfällen kann auch eine Dauer von 12 Wochen notwendig sein [4]. Die Behandlung sollte stets in Kombination mit einer topischen Therapie erfolgen. Hier zeigen insbesondere Ciclopirox und Clotrimazol synergistische Effekte [9].

Eine besondere Herausforderung stellt zudem die Behandlung von Schwangeren dar. Im Bereich der topischen Antimykotika sind insbesondere Clotrimazol und Miconazol zu empfehlen. Systemische Antimykotika sind generell kontraindiziert und können nur im Einzelfall nach ausführlicher Nutzen-Risiko-Abwägung diskutiert werden [10].
